# One Step Closer to Conversational Medical Records: ChatGPT Parses Psoriasis Treatments from EMRs

**DOI:** 10.3390/jcm14217845

**Published:** 2025-11-05

**Authors:** Jonathan Shapiro, Mor Atlas, Sharon Baum, Felix Pavlotsky, Aviv Barzilai, Rotem Gershon, Romi Gleicher, Itay Cohen

**Affiliations:** 1 Maccabi Healthcare Services, Ramat Hasharon 4702633, Israel; 2 Ono Academic, Business Administration Faculty, One Academic Boulevard, Kiryat Ono 5510701, Israel; 3 Dermatology Department, Sheba Medical Center, Ramat-Gan 5262000, Israel; 4 Gray Faculty of Medicine, Tel-Aviv University, Tel-Aviv 6997801, Israel; 5 Ruth and Bruce Rappaport Faculty of Medicine, Technion—Institute of Technology, Haifa 3200003, Israel; 6 Rutgers School of Public Health, Rutgers University, Piscataway, NJ 08854, USA

**Keywords:** artificial intelligence, psoriasis, ChatGPT, electronic medical records

## Abstract

**Background**: Large Language Models (LLMs), such as ChatGPT, are increasingly applied in medicine for summarization, clinical decision support, and diagnostic assistance, including recent work in dermatology. Previous AI and NLP models in dermatology have mainly focused on lesion classification, diagnostic support, and patient education, while extracting structured treatment information from unstructured dermatology records remains underexplored. We evaluated ChatGPT-4o’s ability to identify psoriasis treatments from free-text documentation, compared with expert annotations. **Methods**: In total, 94 electronic medical records (EMRs) of patients diagnosed with psoriasis were analyzed. ChatGPT-4o extracted treatments used for psoriasis from each unstructured clinical note. Its output was compared to manually curated reference annotations by expert dermatologists. A total of 83 treatments, including topical agents, systemic medications, biologics, phototherapy, and procedural interventions, were evaluated. Performance metrics included recall, precision, F1-score, specificity, accuracy, Cohen’s Kappa, and Area Under the Curve (AUC). Analyses were conducted at the individual-treatment level and grouped into pharmacologic categories. **Results**: ChatGPT-4o demonstrated strong performance, with recall of 0.91, precision of 0.96, F1-score of 0.94, specificity of 0.99, and accuracy of 0.99. Agreement with expert annotations was high (Cohen’s Kappa = 0.93; AUC = 0.98). Group-level analysis confirmed these results, with the highest performance in biologics and methotrexate (F1 = 1.00) and lower recall in categories with vague documentation, such as systemic corticosteroids and antihistamines. **Conclusions**: Our study highlights the potential of LLMs to extract psoriasis treatment information from unstructured clinical documentation and structure it for research and decision support. The model performed best with well-defined, commonly used treatments.

## 1. Introduction

The integration of large language models (LLMs), such as ChatGPT, into clinical informatics has opened promising avenues in medical documentation, decision support, and research.

While NLP- and AI-based models have been applied in dermatology for diagnostic assistance, lesion description, and educational simulation, large language models have also shown capabilities in automating administrative tasks, summarizing patient information, and enhancing clinician workflows and patient communication [1,2,3,4,5,6,7,8]. However, their ability to extract and structure detailed treatment histories from unstructured dermatology EMRs, particularly in chronic diseases such as psoriasis, remains underexplored. This gap limits the potential of large language models to support retrospective analyses, treatment pattern recognition, and decision-support systems in dermatology.

Psoriasis is a chronic, immune-mediated skin disease affecting approximately 3% of the global population. Its management involves individualized, often complex regimens, including topical therapies, systemic medications, biologics, phototherapy, and procedural interventions [2,9]. Given the disease’s unpredictable course, risk of progression to psoriatic arthritis, and variability in treatment response, tracking longitudinal treatment history is both clinically essential and operationally challenging. While this information is often documented in electronic medical records (EMRs), it typically resides in free-text form, which lacks standardization and is challenging to analyze retrospectively [8,9,10].

Recent advances in natural language processing (NLP) have enabled AI-powered tools to enhance electronic medical records (EMR) abstraction, with studies showing improved disease identification when narrative text is analyzed alongside structured codes, outperforming code-only algorithms in diseases like psoriatic arthritis and inflammatory dermatoses [9,11]. In dermatology, tools like ChatGPT have demonstrated potential in patient education, clinical summarization, and simulation of board exam scenarios [1,3]. Specifically in psoriasis, ChatGPT-4 has been used to identify affected body areas and comorbidities from clinical narratives [12], with some success in comparing treatment options and supporting patient engagement, although diagnostic limitations remain [2,3,4,5]. In rheumatology, NLP-based classification of psoriatic arthritis has outperformed rule-based models, further supporting the feasibility of LLMs in treatment-related tasks [9].

Clinical documentation, particularly in dermatology EMRs and especially for psoriasis, poses unique linguistic challenges. These include variable terminology, abbreviations, spelling inconsistencies, and linguistic ambiguity. In addition, distinguishing between treatments for psoriasis and those prescribed for comorbid conditions adds further complexity [13,14]. Phrases involving negation or uncertainty (e.g., “the patient was not treated with methotrexate”) must also be interpreted correctly to avoid false positives [15,16].

LLMs like GPT-4 have shown promise in overcoming similar challenges in other domains. For example, they have demonstrated high recall in extracting findings from radiology reports (99.3%), supporting the feasibility of automating data extraction tasks [17]. They have also been effective in de-identifying clinical notes and generating synthetic data, thereby improving administrative workflows and data quality [14,18,19]. In oncology, ChatGPT-4 has been used to identify cancer phenotypes from EHR text, further supporting its value in extracting clinical information from unstructured narratives [20]. Moreover, ChatGPT-4 has achieved diagnostic accuracy comparable to that of physicians when identifying final diagnoses from differential lists [21]

These findings suggest strong potential for LLMs to support automated extraction of structured treatment data from complex, unstructured text, a critical need in dermatology, where documentation practices are exceptionally heterogeneous.

In this exploratory study, we evaluate the general-purpose language model ChatGPT-4o’s ability to extract treatment information from unstructured EMRs of patients with psoriasis.

ChatGPT-4o was selected for this study because it represents one of the most advanced and publicly accessible general-purpose LLMs, with demonstrated high performance in extracting medical information from free-text clinical notes in other domains [22]. Evaluating such a general model, rather than a domain-specific or fine-tuned alternative, allows assessment of baseline feasibility for AI-assisted data extraction using tools that are readily available to clinicians and researchers. By comparing its outputs to gold-standard annotations from expert dermatologists, we assess its accuracy and explore its potential utility in dermatology workflows. This work contributes to the growing body of literature supporting the use of LLMs in dermatology. It aims to inform scalable AI-assisted solutions for clinical documentation, retrospective cohort assembly, and decision support in chronic dermatoses.

## 2. Materials and Methods

### 2.1. Data Collection

We retrospectively reviewed 94 electronic medical records (EMRs) of patients diagnosed with psoriasis and treated at the Dermatology and Psoriasis Clinic at Sheba Medical Center. The EMRs were written in a hybrid of Hebrew and English and included patient anamnesis, physical examination findings, treatment history, and management plans.

### 2.2. Annotation and Standardization

The dataset was annotated by a senior dermatologist and subsequently reviewed by another senior dermatologist to confirm that no treatments were missed and that no annotation errors occurred. The purpose of this secondary review was quality assurance, aimed at verifying completeness and accuracy rather than assessing inter-rater agreement, since disagreements between reviewers were not expected. The finalized annotation dataset served as the gold-standard reference for model evaluation. Each record was manually reviewed to annotate all treatments specifically administered for psoriasis, ensuring that only psoriasis-related therapies were included in the analysis. Treatments documented for other dermatologic conditions were ignored. Each patient case was transformed into a structured dataset entry, with binary indicator variables corresponding to 83 possible treatments. Each treatment was labeled TRUE if used to treat psoriasis and FALSE otherwise, yielding a sparse matrix.

### 2.3. Model Configuration

To evaluate automated extraction, we applied ChatGPT-4o, a general-purpose multimodal language model developed by OpenAI. A custom GPT agent was created on OpenAI’s platform (psoriasis-treatment-extractor), with the following exact instructions: ‘The GPT will read a mix of Hebrew and English summaries provided by the user, extracting only the treatments related to psoriasis. If a treatment is mentioned, the GPT will identify whether the patient received it, note the patient’s response to the treatment, and specify the duration or number of treatment courses if mentioned. The GPT will avoid mentioning treatments for other diseases and focus solely on psoriasis-related information. Mention only treatments in the past and not treatments planned for the future.’ The model was not provided with a predefined medication list and relied solely on the textual content of each EMR. Each note was entered individually in a new session, with the model instructed not to save any data for future training. The extracted treatments were then manually copied into a binary-coded table identical in structure to the expert-annotated dataset.

### 2.4. Evaluation Metrics

For model evaluation, we obtained parallel binary classifications, one from the human expert and one from ChatGPT-4o, for each of the 94 records and 83 treatments. To assess model performance, we calculated accuracy, precision, recall, F1-score, specificity, Cohen’s Kappa, and the area under the receiver operating characteristic curve (AUC).

All treatments were standardized to their generic names during annotation and analysis. Brand names appearing in EMRs (e.g., Dermovate) were mapped to their generic equivalents (clobetasol propionate) to ensure terminological consistency. Specifically, “Topical ointments” reflects the exact wording documented by the dermatologist in the electronic medical record in some cases. In these cases, the treatment was recorded simply as “Topical ointments” without further specification, and this terminology was preserved in the analysis to maintain fidelity to the original clinical documentation. The vehicle used in topical therapies (ointment, cream, foam, gel, solution, etc.) was not always explicitly documented in the EMRs. In most cases, the vehicle was clinically apparent from the clinical context, and for several commonly used products (e.g., Xamiol), only a single marketed vehicle exists, making the vehicle unambiguous. For other topical corticosteroids available in multiple vehicles under the same brand name, the vehicle used was sometimes documented and sometimes not. Because in the vast majority of cases the vehicle could be inferred, we did not design a separate sub-analysis for vehicle extraction.

Treatment categories were defined with pharmacologic precision (e.g., topical corticosteroids, systemic corticosteroids, intralesional corticosteroids) to reflect route of administration.

One treatment category, ‘Unspecified topical treatments,’ aggregates all cases in which a topical therapy was documented without specifying the product name or vehicle, allowing consistent classification despite incomplete documentation.

### 2.5. Statistical Analysis

Statistical evaluation was conducted at multiple levels: (1) a global level, treating each treatment-patient combination as a separate binary instance (7802 in total); (2) treatment-wise, where each treatment was evaluated as a separate classification task (limited to treatments with at least five positive cases); and (3) group-level, where treatments were clustered into pharmacologic categories (e.g., biologics, topical corticosteroids) to increase statistical stability and interpretability. Table 1 outlines the pharmacologic categories and their corresponding treatments used to group medications for the group-level analysis. Lastly, we computed per-record Cohen’s Kappa to assess agreement on a patient-level basis.

All analyses were conducted using R (version 4.4.1).

IRB approval status: Reviewed and approved by the Sheba Medical Center Ethics Committee, approval number 1083-24-SMC.

Declaration of generative AI and AI-assisted technologies in the writing process: During the preparation of this work, the author used ChatGPT-4o to improve language and readability. After using this tool, the author reviewed and edited the content as needed and takes full responsibility for the content of the publication.

## 3. Results

Of the 94 psoriasis cases included in the study, 55 were female (58.5%) and 39 were male (41.5%). The age range of patients was 18.9 to 86.7 years. Clinical visit notes averaged 278 ± 154 words in length. The number of psoriasis treatments per patient varied from zero (in five cases) to twelve, with a median of three and a mean of 3.2. Some treatments, including Loratadine, Dexacort, and IV Experimental therapy, were rare and were missed by the model. Conversely, ChatGPT-4o misidentified three treatments as psoriasis-related that were actually indicated for other conditions.

At the global level, ChatGPT-4o demonstrated strong overall performance across 7802 binary instances (83 treatments × 94 records). It achieved a recall of 0.91 and a precision of 0.96, resulting in an F1-score of 0.94. Specificity and accuracy both reached 0.99, and Cohen’s Kappa was 0.93, indicating strong agreement with expert annotations beyond chance. The model’s AUC was 0.98, reflecting excellent discrimination between prescribed and non-prescribed treatments.

**Figure 1:**
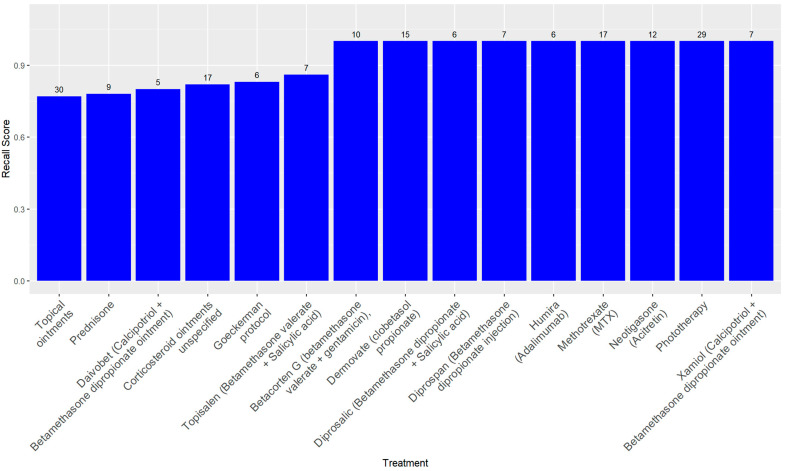
Recall score of treatment with more than five positive cases. The number on top of each column represents the number of positive cases in the database.

A treatment-wise evaluation, limited to treatments administered in at least five patient records, confirmed these trends. Perfect precision (1.00) was observed across nearly all treatments, indicating a low false positive rate. Recall values were more variable, ranging from 0.77 for treatments such as unspecified topical ointments to 1.00 for well-documented therapies including Adalimumab (Humira), MTX, Phototherapy, and Clobetasol propionate (Dermovate). The F1-score exceeded 0.90 for most treatments. Specificity was consistently near 1.00. Cohen’s Kappa values ranged from 0.79 to 1.00, with most above 0.86, and AUC scores were uniformly strong, all exceeding 0.88. Table 2 presents the full set of performance metrics for these treatments, while Figure 1 illustrates their respective recall values.

**Figure 2:**
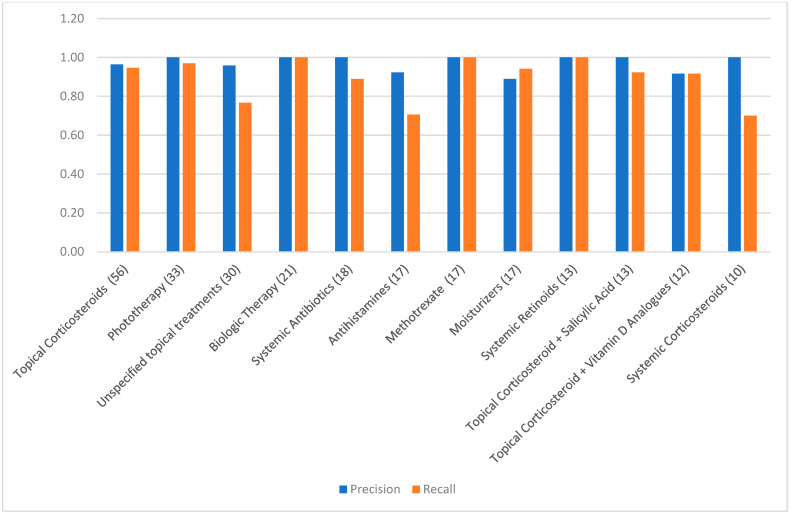
Group-level precision and recall.

To enhance statistical stability, a group-level analysis was performed by aggregating treatments into pharmacologic categories. We included 15 of 23 possible categories, excluding those with fewer than five total observations. Categories included biologics, topical corticosteroids, systemic antibiotics, antihistamines, and systemic retinoids. ChatGPT-4o maintained excellent precision across all included groups, ranging from 0.85 to 1.00. Recall values ranged from 0.70 (e.g., systemic corticosteroids, antihistamines) to 1.00 (e.g., biologics, MTX, salicylic acid preparations), with most groups exceeding 0.89. Group-level F1-scores were generally ≥0.90, and specificity and accuracy remained high. AUC values consistently exceeded 0.92 across all categories. Cohen’s Kappa was above 0.90 in nearly all groups. Table 3 presents the complete set of performance metrics for each treatment category. Figure 2 provides a visual comparison of precision and recall values across categories with 10 or more observations. Despite variability in recall for a few categories, particularly systemic corticosteroids and antihistamines, the model’s precision and agreement with expert annotation remained consistently high. These findings underscore ChatGPT-4o’s strong performance in extracting real-world psoriasis treatments across diverse pharmacologic domains and documentation styles.

## 4. Discussion

The current era of healthcare is marked by unprecedented growth in clinical data, both in volume and in complexity. Electronic medical records (EMRs), originally introduced to streamline documentation and improve continuity of care, have paradoxically created new burdens on clinicians. Numerous studies have highlighted that physicians now spend more time entering data into EMRs than they do in face-to-face patient encounters, with documentation often extending beyond clinical hours and contributing substantially to professional burnout [23,24]. This phenomenon, sometimes referred to as “pajama time”, highlights how the promise of digitization has not yet fully materialized in everyday practice. Instead of improving efficiency, EMRs have frequently added layers of administrative work that detract from clinical care.

The development and integration of artificial intelligence (AI) systems capable of parsing unstructured medical text and generating structured visit summaries is not merely a technological innovation but a systemic necessity. Healthcare systems worldwide are grappling with rising patient volumes, workforce shortages, and escalating costs. These pressures underscore the need for scalable solutions that can reduce administrative burden while simultaneously improving the fidelity of medical data. In this context, large language models (LLMs) such as ChatGPT represent a paradigm shift: rather than asking clinicians to adapt their documentation practices to rigid data-entry forms, AI systems can adapt to clinicians’ natural language, extracting key details with high precision and recall [25].

Furthermore, transforming free-text clinical narratives into structured, analyzable data is foundational to contemporary healthcare initiatives such as value-based care, population health management, and precision medicine. Longitudinal tracking of therapies, treatment responses, and adverse events is indispensable for optimizing individual patient outcomes and for generating real-world evidence at scale. Traditionally, these tasks have relied on labor-intensive chart review and manual data abstraction, constraining both efficiency and scope. By automating the conversion of narrative documentation into structured treatment histories, AI-driven systems can unlock vast repositories of previously inaccessible information, enabling rapid cohort identification, large-scale retrospective analyses, and data-driven decision support across diverse clinical settings [26].

It is important to situate this transformation within a broader technological trajectory. The past decade has witnessed incremental advances in natural language processing applied to clinical documentation, ranging from rule-based systems to statistical models and domain-specific machine learning approaches. Each stage improved performance but remained constrained by brittle rules or narrow vocabularies. The emergence of general-purpose LLMs, trained in vast multilingual corpora and capable of contextual reasoning, represents a step change in capability. Unlike earlier systems, these models are not limited to recognizing predefined keywords or phrases but can interpret ambiguous descriptions, handle linguistic variability, and infer clinical relevance across diverse contexts. This allows them to engage with EMRs in a manner that is far closer to human clinical reasoning, bridging the gap between free-text notes and structured data repositories [27].

Beyond efficiency and accuracy, the integration of AI into clinical documentation has broader implications for the physician–patient relationship and the future of medical practice. By reducing the time spent on clerical tasks, AI-assisted systems have the potential to restore clinicians’ focus to direct patient care, improving satisfaction on both sides of the clinical encounter [22]. At the same time, the ability to generate comprehensive, accurate, and standardized summaries enhances communication among multidisciplinary teams, reduces the risk of missed information, and supports continuity of care across healthcare settings.

From a research standpoint, these developments also herald a new era in the generation of medical knowledge. The ability to query large EMR databases with AI tools enables researchers to identify patient subgroups, treatment patterns, and outcome trajectories at a speed and scale that manual chart review could never achieve. This creates opportunities for rapid hypothesis generation, real-world evidence studies, and post-marketing surveillance, all of which are increasingly recognized as essential complements to randomized controlled trials in guiding clinical decision-making [28].

The adoption of advanced AI tools such as ChatGPT-4o for treatment extraction from EMRs offers significant advantages for both clinical practice and research. In the clinical setting, one of the most promising benefits is the potential to generate comprehensive summaries of a patient’s treatment history with reduced effort and improved accuracy. Traditionally, compiling such summaries during patient intake or follow-up visits requires clinicians to manually sift through lengthy and often fragmented medical records, a process that is both time-consuming and prone to error. While the current use of AI-powered extraction does not yet result in substantial time savings, it introduces a structured and consistent approach to retrieving relevant treatments, which may reduce the risk of missing critical information [12,14,29]. As technology continues to evolve, it may also lead to meaningful time savings in clinical workflows. Moreover, once generative AI models can consistently extract treatments with high precision, EMRs can serve as contextual inputs for interactive models, enabling the use of clinical notes as file-based data sources in a Retrieval-Augmented Generation (RAG) framework, forming the basis for informed, case-specific conversations with an AI assistant.

For research, the implications are equally profound. AI models can be leveraged to perform large-scale cohort identification and retrospective analyses that would be infeasible with manual review. For instance, researchers can efficiently query the EMR database to identify all patients who did not respond to methotrexate (MTX) or who have been treated with a specific medication, supporting studies of treatment effectiveness, safety, and real-world outcomes [14,20]. This capability accelerates the pace of clinical research, enables rapid hypothesis generation and testing, and facilitates the development of precision medicine approaches by uncovering nuanced patterns in treatment response across diverse patient populations. As these tools continue to evolve, their integration into clinical and research workflows promises to enhance the quality, efficiency, and impact of both patient care and medical discovery.

This study explored ChatGPT-4o’s ability to accurately identify therapies associated with psoriasis from complex, multilingual (Hebrew-English hybrid) medical records, distinguish them from treatments for other conditions, and compare its extraction accuracy to that of expert human annotation. The goal determined the feasibility and reliability of integrating advanced AI tools into clinical and research workflows for automated treatment identification in dermatology.

There is an abundance of LLMs available in the AI ecosystem. We chose OpenAI’s GPT because it has the highest market share [30,31]. In addition, this study builds on our previous work with ChatGPT [12]. There are also open-sourced models that can be used, and even further fine-tuned to a specific domain, such as Mistral [32,33]. However, in this work, we aimed to evaluate the performance of a general-purpose model in informing and guiding potential users, focusing on the most commonly used publicly accessible option. It is worth noting that other models may achieve higher accuracy, particularly when trained explicitly on EMRs.

One main challenge in extracting treatment histories from unstructured EMRs is the variability in how treatments are described. In some cases, the documentation includes specific drug names, such as methotrexate or calcipotriol, which the model can more reliably recognize as administered treatments. However, in other instances, the clinical notes refer to general treatment categories, such as “topical treatments,” “topical steroids,” or “biologic therapies”, without naming a particular medication. While these general terms likely indicate actual therapeutic use, they pose a challenge for the model in determining whether and how to classify them as concrete treatments for psoriasis.

In our study, ChatGPT-4o demonstrated high overall performance, with an accuracy of 0.99, a precision of 0.96, a recall of 0.91, and an F1 score of 0.94. Cohen’s Kappa (0.93) and AUC (0.98) further supported its excellent discriminative ability and agreement with expert annotations. Treatment-specific and pharmacologic-group analyses highlighted strong overall metrics, although some variability in recall was observed, particularly for treatments with less explicit or more ambiguous documentation.

While ChatGPT-4o performed robustly in treatment identification, a deeper exploration of groups with lower recall provides important insights into model behavior and highlights areas for refinement. For example, the relatively low recall for systemic corticosteroids and antihistamines, despite their presence in many patient records, corresponds with the lower Cohen’s Kappa values for these treatment groups listed in Table 3 (antihistamines κ = 0.79; systemic corticosteroids κ = 0.77). These findings likely reflect not only model limitations but also clinical and contextual ambiguity. Systemic corticosteroids are generally not recommended for psoriasis due to the risk of rebound flares, and their use is typically confined to atypical cases or early diagnostic workups. In several instances, patients may have received systemic corticosteroids before a definitive diagnosis of psoriasis was established, making it technically correct to list them as part of the treatment history, but potentially confusing for the model, which lacks the ability to infer temporal context or diagnostic intent. Similarly, antihistamines are often prescribed to patients with psoriasis suffering from severe pruritus, yet they are not disease-modifying agents and may be recorded as general symptomatic treatments. This variability in clinical context, coupled with nonspecific phrasing in electronic medical records (EMRs), likely contributed to the model’s reduced ability to identify these therapies as psoriasis-related treatments consistently. In other instances, ChatGPT-4o identified medications that the human investigator did not explicitly mention.

A detailed examination of these lower-performing cases revealed several distinct patterns: One notable source of error was ChatGPT-4o incorrectly interpreting planned future treatments as treatments already administered. This misunderstanding primarily stemmed from ambiguities in clinical documentation, where future treatment plans were not clearly distinguished from historical or ongoing treatments. While this distinction is clear to human clinicians due to context or clinical familiarity, ChatGPT-4o occasionally struggled with this nuance. This emphasizes the need for more explicit differentiation within clinical notes between historical, current, and future treatment plans to enhance NLP accuracy. However, as the models improve, we believe their ability to distinguish past treatments from recommendations will also improve.

An interesting and proactive behavior exhibited by ChatGPT-4o was its holistic approach to psoriasis patient management. ChatGPT-4o occasionally suggested medications not specifically annotated by the human reviewer as psoriasis treatments but identified them as potential therapies due to their management of known comorbidities associated with psoriasis. For instance, ChatGPT-4o spontaneously listed statins used for treating hypercholesterolemia as potentially relevant treatments, justifying their inclusion by citing the well-established association between psoriasis and metabolic syndrome. Similarly, ChatGPT-4o independently considered psychiatric medications prescribed for depression as related to psoriasis management, due to the documented association between psoriasis and depression.

Furthermore, ChatGPT-4o proactively identified in some cases antifungal treatments prescribed empirically for suspected tinea pedis, reasoning that these treatments could be relevant in cases where clinicians might face diagnostic ambiguity between psoriasis and fungal infections or when treating fungal infections complicating psoriasis plaques. GPT-4o provided these additional interpretations with explanatory remarks, allowing human reviewers to explicitly determine their relevance.

Other medications proactively listed by ChatGPT-4o included liraglutide (Saxenda) for obesity management, reflecting careful consideration of obesity as an important comorbidity that dermatologists closely monitor in patients with psoriasis. ChatGPT-4o also noted medical cannabis usage, expressing appropriate reservations about its explicit classification as a psoriasis treatment but nevertheless flagging its potential clinical relevance.

These proactive inclusions by ChatGPT-4o highlight the sophisticated, clinically informed reasoning that AI models can perform, surpassing the strict labeling provided by human reviewers. However, this also suggests the necessity of clear instructions in AI prompts regarding whether to include or explicitly exclude treatments for psoriasis-associated comorbidities, depending on the intended clinical or research context. For research purposes, this ability may lead to more insights into treatment options and patterns.

Another practical reason for treatment misidentification observed was ChatGPT-4o’s occasional misreading or incorrect interpretation of medication names. For instance, ChatGPT-4 mistakenly read “Dermovate” (clobetasol propionate) instead of “Daivobet” (Calcipotriol + Betamethasone dipropionate), both spelled in Hebrew, a misinterpretation that highlights the inherent limitations of AI in recognizing medication names. This demonstrates that, despite overall impressive accuracy, the model can still make specific identification errors, reinforcing the continued necessity of human oversight. These types of errors are also expected to decrease with more training and model improvements.

The importance of standardizing drug nomenclature in EMRs is underscored by the variability between brand and generic names and by vague terms such as ‘steroid’ which can hinder both manual annotation and AI-based extraction. By mapping brand names to generic equivalents and defining pharmacologic categories precisely, greater consistency and interpretability were achieved.

This work should be viewed within a broader transformation taking place in medicine. Across specialties, AI tools are being developed to analyze patient records and generate structured summaries for both clinical practice and research. Our study demonstrates how this vision can be operationalized: using a general-purpose language model, we showed that treatment information embedded in dermatology notes can be reliably extracted and organized. Although psoriasis was chosen as an initial test case, the approach extends to other chronic diseases where treatment histories are equally complex and clinically significant. By demonstrating that a non-specialized LLM can achieve high accuracy in parsing treatments from routine EMRs, this work provides proof of concept for scalable AI-assisted medical record summarization. Such capabilities have the potential to reduce clinicians’ documentation burden, strengthen continuity of care, and unlock new opportunities for real-world data analysis across the medical field.

## 5. Limitations

Our study had several limitations. First, due to the limited number of positive instances for several treatment labels, the statistical analysis is primarily descriptive. Future research with a substantially larger dataset is needed to more reliably assess the model’s sensitivity in identifying different treatments.

Second, in our analysis, we did not differentiate between topical therapy vehicle types (e.g., ointment, cream, gel, foam, solution). When the AI and the human reviewer both identified the same brand name, it was counted as a match regardless of discrepancies in vehicle specifications. This simplified matching strategy may overlook clinically meaningful differences when the same brand is marketed in multiple formulations, potentially overestimating agreement between AI and human outputs. Future work should incorporate sub-analyses by vehicle type to identify discrepancies between what is documented in the EMR and what the AI extracts. Beyond retrospective analysis, this could also enable real-time clinical decision support: for example, prompting physicians to specify the vehicle type when documenting brand names associated with multiple vehicles, thereby improving data quality and clinical interpretability.

Third, ChatGPT-4o was not pre-trained on a predefined list of psoriasis treatments. Although overall performance was high, we expect that results would further improve with fine-tuning on domain-specific treatment vocabularies.

Fourth, the unstructured EMR data consisted of a mix of Hebrew and English. We assume that performance on fully English-based EMRs would likely be higher.

Fifth, this study has a relatively modest sample size of 94 records. Although the dataset generated more than 7800 binary treatment instances, many treatments were represented by only a handful of times, which constrained statistical power and reduced the stability of per-treatment performance metrics. To validate these findings and improve robustness, larger multi-center datasets will be necessary, enabling greater generalizability across diverse populations and documentation practices.

Finally, in most of the electronic medical records, treatments were documented retrospectively, and the distinction between past and current therapies was not always explicit, making it difficult even for human reviewers to determine which treatments were ongoing at the time of documentation. When future or planned treatments were mentioned, it was often unclear whether such treatments were eventually initiated by the patient. Therefore, this exploratory study focused on identifying treatments that were clearly documented as having been administered. Future work should expand this approach to include explicit temporal classification and the reconstruction of treatment timelines, thereby enabling evaluation of the model’s ability to interpret longitudinal treatment information. Future research may also explore the use of fine-tuned language models trained specifically on dermatologic corpora to enhance context recognition and extraction accuracy.

## Figures and Tables

**Table 1:** Pharmacologic Categories and Included Treatments for Psoriasis.

Pharmacologic Group	Included Treatments: As They Appear in the EMR’s Free Text (Generic Name)
Topical Corticosteroids (TCS)	Elocom (mometasone furoate), Aflumycin (Prednisolone + Gentamicin), Betacorten G (betamethasone valerate + gentamicin), Dermovate (clobetasol propionate), Dermacombin (Triamcinolone + Gramicidin + Nystatin + Neomycin), nonspecific corticosteroid ointments, Neriderm (Diflucortolone Valerate), Fucicort (betamethasone valerate + fusidic acid), Threolone (Prednisolone + Chloramphenicol), Diprozone (betamethasone dipropionate), Tevaderm (Diflucortolone valerate + Isoconazole)
Topical Corticosteroid + Vitamin D Analogues	Daivobet (Calcipotriol + Betamethasone dipropionate ointment), Xamiol (Calcipotriol + Betamethasone dipropionate Gel
Topical Corticosteroid + Salicylic Acid	Diprosalic (Betamethasone dipropionate + Salicylic acid), Topisalen (Betamethasone valerate + Salicylic acid)
Salicylic Acid	Salicylic acid preparations, Salikaren (Salicylic acid 3%)
TAR-based Topicals	Goeckerman protocol, TAR ointments
Systemic Retinoids	Neotigasone (Acitretin)
Methotrexate (MTX)	MTX, Methotrexate
Biologic Therapy	Humira (Adalimumab), Cosentyx (Secukinumab), Enbrel (Etanercept), Skyrizi (Risankizumab), Stelara (Ustekinumab), Taltz (Ixekizumab), Remicade (Infliximab), Simponi, other biologics not individually named
Systemic Corticosteroids	Prednisone, Dexacort (Dexamethasone)
Corticosteroids—Intralesional (IL)	Diprospan (Betamethasone dipropionate injection)
Phototherapy	Phototherapy (UV), Goeckerman protocol
Antihistamines	Ahiston (Chlorpheniramine Maleate), Allergix (Loratadine), Aerius (desloratadine), Telfast (fexofenadine), Loratadine (loratadine), Lorastin (loratadine), Phenergan (promethazine), Atarax (hydroxyzine), unspecified antihistamines
Systemic Antibiotics	Augmentin PO (Amoxicillin + Clavulanic acid), others not explicitly named
Moisturizers	Emollients, unspecified moisturizers, Aqua cream, Dermacol, U lactin, Sebocalm, Vaseline, Cetomacrogol

With or without antibiotic or antifungal agents.

**Table 2:** Per-treatment performance metrics for treatments with ≥5 positive Instances.

Treatment	Precision	Recall	F1	SP	AU	AC	KP	TP	TN	FP	FN	Positive Cases
Adalimumab (Humira)	1	1	1	1	1	1	1	6	88	0	0	6
Goeckerman protocol	1	0.83 ± 0.30	0.91 ± 0.23	1	0.92	0.99 ± 0.02	0.9	5	88	0	1	6
Methotrexate	1	1	1	1	1	1	1	17	77	0	0	17
Phototherapy	1	1	1	1	1	1	1	29	65	0	0	29
Betamethasone dipropionate + Salicylic acid (Diprosalic)	1	1	1	1	1	1	1	6	88	0	0	6
Betamethasone valerate + Salicylic acid (Topisalen)	1	0.86 ± 0.26	0.92 ± 0.20	1	0.93	0.99 ± 0.02	0.92	6	87	0	1	7
Calcipotriol + Betamethasone dipropionate (Xamiol)	1	1	1	1	1	1	1	7	87	0	0	7
Betamethasone sodium phosphate + Betamethasone dipropionate (Diprospan)	1	1	1	1	1	1	1	7	87	0	0	7
Prednisone	1	0.78 ± 0.27	0.88 ± 0.22	1	0.89	0.98 ± 0.03	0.86	7	85	0	2	9
Betamethasone + Gentamicin (Betacorten G)	1	1	1	1	1	1	1	10	84	0	0	10
Clobetasol propionate (Dermovate)	0.88 ± 0.15	1	0.94 ± 0.12	0.97 ± 0.03	0.99	0.98 ± 0.03	0.92	15	77	2	0	15
Corticosteroid ointments	1	0.82 ± 0.18	0.90 ± 0.14	1	0.91	0.97 ± 0.04	0.88	14	77	0	3	17
Topical ointments	0.96 ± 0.08	0.77 ± 0.15	0.85 ± 0.13	0.98 ± 0.03	0.88	0.91 ± 0.06	0.79	23	63	1	7	30
Acitretin (Neotigasone)	1	1	1	1	1	1	1	12	82	0	0	12
Calcipotriol + Betamethasone dipropionate (Daivobet)	0.80 ± 0.35	0.80 ± 0.35	0.80 ± 0.35	0.99 ± 0.02	0.89	0.98 ± 0.03	0.79	4	88	1	1	5

SP—Specificity, AU—Area Under the Curve, AC—Accuracy, KP = Kappa, TP—True Positive, TN—True Negative, FP—False Positive, FN—False Negative.

**Table 3:** Group-level Treatment Performance Metrics for groups with more than five positive cases. Medication represents the number of treatments in the group.

Group	MD	N	Pre	Recall	F1	SP	AUC	AC	Kappa	TP	TN	FP	FN
Antibiotics–systemic	8	18	1	0.89 ± 0.15	0.94 ± 0.11	1	0.94	1.00 ± 0.00	0.94	16	734	0	2
Anti–histamines	9	17	0.92 ± 0.14	0.71 ± 0.22	0.80 ± 0.19	1.00 ± 0.00	0.85	0.99 ± 0.01	0.8	12	828	1	5
Biologic therapy	8	21	1	1	1	1	1.00	1	1	21	731	0	0
Goeckerman	1	6	1	0.83 ± 0.30	0.91 ± 0.23	1	0.92	0.99 ± 0.02	0.9	5	88	0	1
Moisturizers	8	17	0.89 ± 0.15	0.94 ± 0.11	0.91 ± 0.36	1.00± 0.01	0.97	1	0.91	16	733	2	1
MTX	1	17	1	1	1	1	1.00	1	1	17	77	0	0
Phototherapy	2	33	1	0.97 ± 0.06	0.98± 0.04	1	0.98	0.99 ± 0.01	0.98	32	155	0	1
Salicylic acid	2	6	0.86 ± 0.26	1	0.92 ± 0.21	0.99 ± 0.01	1.00	0.99 ± 0.01	0.92	6	181	1	0
Topical Corticosteroids + Salicylic acid	2	13	1	0.92 ± 0.14	0.96 ± 0.11	1	0.96	0.99 ± 0.01	0.96	12	175	0	1
Topical Corticosteroids + vitamin D	2	12	0.92 ± 0.16	0.92 ± 0.16	0.92 ± 0.16	0.99 ± 0.01	0.96	0.99 ± 0.01	0.91	11	175	1	1
Corticosteroids–Intralesional (IL)	1	7	1	1	1	1	1.00	1	1	7	87	0	0
Corticosteroids–systemic	2	10	1	0.70 ± 0.28	0.82 ± 0.24	1	0.85	0.98 ± 0.02	0.82	7	178	0	3
Systemic retinoids	2	13	1	1	1	1	1.00	1	1	13	175	0	0
TAR–topical	3	8	0.89 ± 0.21	1	0.94 ± 0.16	1.00 ± 0.01	1.00	1.00 ± 0.01	0.94	8	273	1	0
TCS (Topical Corticosteroids)	12	56	0.96 ± 0.04	0.95 ± 0.06	0.95 ± 0.15	1.00 ± 0.00	0.98	1	0.95	53	1070	2	3

MD—Medication, N— Number of cases, Pre—Precision, SP—Specificity, AUC—Area Under the Curve, AC—Accuracy, TP—True Positive, TN—True Negative, FP—False Positive; FN—False Negative.

## Data Availability

The data supporting the findings of this study are available from the corresponding author upon request, owing to privacy/ethical restrictions.

## References

[1] Jin J.Q., Dobry A.S. (2023). ChatGPT for healthcare providers and patients: Practical implications within dermatology. J. Am. Acad. Dermatol..

[2] Ravipati A., Elman S.A. (2024). The state of artificial intelligence for systemic dermatoses: Background and applications for psoriasis, systemic sclerosis, and much more. Clin. Dermatol..

[3] Goktas P., Grzybowski A. (2024). Assessing the Impact of ChatGPT in Dermatology: A Comprehensive Rapid Review. J. Clin. Med..

[4] Khamaysi Z., Awwad M., Jiryis B., Bathish N., Shapiro J. (2025). The Role of ChatGPT in Dermatology Diagnostics. Diagnostics.

[5] Shapiro J., Avitan-Hersh E., Greenfield B., Khamaysi Z., Dodiuk-Gad R.P., Valdman-Grinshpoun Y., Freud T., Lyakhovitsky A. (2025). The use of a ChatGPT-4-based chatbot in teledermatology: A retrospective exploratory study. J. Dtsch. Dermatol. Ges..

[6] Baker M.N., Burruss C.P., Wilson C.L. (2023). ChatGPT: A Supplemental Tool for Efficiency and Improved Communication in Rural Dermatology. Cureus.

[7] Shah A., Wahood S., Guermazi D., Brem C.E., Saliba E. (2024). Skin and Syntax: Large Language Models in Dermatopathology. Dermatopathology.

[8] Paganelli A., Spadafora M., Navarrete-Dechent C., Guida S., Pellacani G., Longo C. (2024). Natural language processing in dermatology: A systematic literature review and state of the art. J. Eur. Acad. Dermatol. Venereol..

[9] Love T.J., Cai T., Karlson E.W. (2011). Validation of psoriatic arthritis diagnoses in electronic medical records using natural language processing. Semin. Arthritis Rheum..

[10] Ford E., Carroll J.A., Smith H.E., Scott D., Cassell J.A. (2016). Extracting information from the text of electronic medical records to improve case detection: A systematic review. J. Am. Med. Inform. Assoc..

[11] Perrin J., Petronic-Rosic V. (2024). The potential role and restrictions of artificial intelligence in medical school dermatology education. Clin. Dermatol..

[12] Shapiro J., Baum S., Pavlotzky F., Mordechai Y.B., Barzilai A., Freud T., Gershon R. (2024). Application of an NLP AI Tool in Psoriasis: A Cross-Sectional Comparative Study on Identifying Affected Areas in Patients’ Data. Clin. Dermatol..

[13] Bodenreider O. (2008). Biomedical ontologies in action: Role in knowledge management, data integration and decision support. Yearb. Med. Inform..

[14] Wang Y., Wang L., Rastegar-Mojarad M., Moon S., Shen F., Afzal N., Liu S., Zeng Y., Mehrabi S., Sohn S. (2018). Clinical information extraction applications: A literature review. J. Biomed. Inform..

[15] WChapman W., Bridewell W., Hanbury P., Cooper G.F., Buchanan B.G. (2001). A simple algorithm for identifying negated findings and diseases in discharge summaries. J. Biomed. Inform..

[16] Sohn S., Wagholikar K.B., Li D., Jonnalagadda S.R., Tao C., Elayavilli R.K., Liu H. (2013). Comprehensive temporal information detection from clinical text: Medical events, time, and TLINK identification. J. Am. Med. Inform. Assoc..

[17] Hasani A.M., Singh S., Zahergivar A., Ryan B., Nethala D., Bravomontenegro G., Mendhiratta N., Ball M., Farhadi F., Malayeri A. (2024). Evaluating the performance of Generative Pre-trained Transformer-4 (GPT-4) in standardizing radiology reports. Eur. Radiol..

[18] Altalla B., Abdalla S., Altamimi A., Bitar L., Al Omari A., Kardan R., Sultan I. (2025). Evaluating GPT models for clinical note de-identification. Sci. Rep..

[19] Huang J., Yang D.M., Rong R., Nezafati K., Treager C., Chi Z., Wang S., Cheng X., Guo Y., Klesse L.J. (2024). A critical assessment of using ChatGPT for extracting structured data from clinical notes. npj Digit. Med..

[20] Bhattarai K., Oh I.Y., Sierra J.M., Tang J., Payne P.R.O., Abrams Z., Lai A.M. (2024). Leveraging GPT-4 for identifying cancer phenotypes in electronic health records: A performance comparison between GPT-4, GPT-3.5-turbo, Flan-T5, Llama-3-8B, and spaCy’s rule-based and machine learning-based methods. JAMIA Open.

[21] Nori H., McKinney S.M., Carignan D., Horvitz E. (2023). Capabilities of GPT-4 on Medical Challenge Problems. arXiv.

[22] Menezes M.C.S., Hoffmann A.F., Tan A.L.M., Nalbandyan M., Omenn G.S., Mazzotti D.R., Hernández-Arango A., Visweswaran S., Venkatesh S., Mandl K.D. (2025). The potential of Generative Pre-trained Transformer 4 (GPT-4) to analyse medical notes in three different languages: A retrospective model-evaluation study. Lancet Digit. Health.

[23] Holmgren A.J., Apathy N.C., Sinsky C.A., Adler-Milstein J., Bates D.W., Rotenstein L. (2025). Trends in Physician Electronic Health Record Time and Message Volume. JAMA Intern. Med..

[24] Tajirian T., Lo B., Strudwick G., Tasca A., Kendell E., Poynter B., Kumar S., Chang P.B., Kung C., Schachter D. (2025). Assessing the Impact on Electronic Health Record Burden After Five Years of Physician Engagement in a Canadian Mental Health Organization: Mixed-Methods Study. JMIR Hum. Factors.

[25] Van Veen D., Van Uden C., Blankemeier L., Delbrouck J.B., Aali A., Bluethgen C., Pareek A., Polacin M., Reis E.P., Seehofnerova A. (2024). Adapted large language models can outperform medical experts in clinical text summarization. Nat. Med..

[26] Bednarczyk L., Reichenpfader D., Gaudet-Blavignac C., Ette A.K., Zaghir J., Zheng Y., Bensahla A., Bjelogrlic M., Lovis C. (2025). Scientific Evidence for Clinical Text Summarization Using Large Language Models: Scoping Review. J. Med. Internet. Res..

[27] Croxford E., Gao Y., Pellegrino N., Wong K., Wills G., First E., Liao F., Goswami C., Patterson B., Afshar M. (2025). Current and future state of evaluation of large language models for medical summarization tasks. npj Health Syst..

[28] Olaker V.R., Fry S., Terebuh P., Davis P.B., Tisch D.J., Xu R., Miller M.G., Dorney I., Palchuk M.B., Kaelber D.C. (2025). With big data comes big responsibility: Strategies for utilizing aggregated, standardized, de-identified electronic health record data for research. Clin. Transl. Sci..

[29] Dobry A., Begaj T., Mengistu K., Sinha S., Droms R., Dunlap R., Wu D., Adhami K., Stavert R. (2021). Implementation and Impact of a Store-and-Forward Teledermatology Platform in an Urban Academic Safety-Net Health Care System. Telemed. e-Health.

[30] Newswire P. (2025). New Statcounter AI Data Finds ChatGPT Sends 79.8% of All Chatbot Referrals to Websites. PR Newswire. https://www.prnewswire.com/news-releases/new-statcounter-ai-data-finds-chatgpt-sends-79-8-of-all-chatbot-referrals-to-websites-302476338.html.

[31] (2025). Statcounter, AI Chatbot Market Share Worldwide, Statcounter Global Stats. https://gs.statcounter.com/ai-chatbot-market-share.

[32] Jiang A.Q.S., Roux A., Mensch A., Savary B., Bamford C., Chaplot D.S., de las Casas D., Hanna E.B., Bressand F. (2024). Mixtral of experts. arXiv.

[33] Randhawa A.R., Zakka C., Hiesinger W., Sattar M. (2024). 7543 Comparative Analysis of Language Model Systems In Endocrinology: Performance And Human Acceptability Assessment. J. Endocr. Soc..

